# Fabrication Water Solubility of Curcumin–Mogroside Self‐Assembly Nanoparticles: Characterization, Antioxidant, and HepG2 Cell Cytotoxicity Activity Evaluation

**DOI:** 10.1155/ijfo/1744361

**Published:** 2025-09-09

**Authors:** Minmin Chen, Fengling Qiu, Youzuo Zhang, Guangzhi Xu

**Affiliations:** ^1^ College of Food and Health, Zhejiang A&F University, Hangzhou, Zhejiang, China, zafu.edu.cn

**Keywords:** curcumin, cytotoxicity, DPPH radical scavenging activity, mogroside, self-assembly nanoparticles, solubility

## Abstract

Curcumin (CUR) is a hydrophobic polyphenol from turmeric with various biological activities. However, its poor water solubility limits its application in food and pharmaceutical fields. Here, the curcumin and mogroside (MOG) self‐assembled nanoparticles (CUR–MOG NPs) with well water solubility were successfully prepared. The CUR–MOG NPs were characterized by dynamic light scattering (DLS), transmission electron microscopy (TEM), x‐ray diffraction (XRD), UV‐visible spectrophotometer, Fourier transform infrared (FTIR) spectroscopy, and thermogravimetric analysis (TGA). The antioxidant activity and HepG2 cell cytotoxicity were further evaluated. CUR–MOG NPs exhibited a particle size of 59.96 ± 0.852 nm with a polydispersity coefficient of 0.077 ± 0.013. The CUR encapsulation efficiency of CUR–MOG NPs was 86.35*%* ± 0.877*%*. The XRD, UV‐visible spectrophotometer, FTIR, and TGA confirmed that the *π*–*π* stacking, hydrophobic interaction, and hydrogen bonding may contribute to the successfully formed self‐assembled nanoparticles. Moreover, nanoparticles had significantly enhanced the water solubility of CUR, increasing from 10.7 ng/mL in pure CUR to 1.135 mg/mL in CUR–MOG NPs. The CUR–MOG NPs demonstrated comparable DPPH radical scavenging activity and HepG2 cell cytotoxicity with those of free CUR. CUR–MOG NPs exhibited a superior water‐soluble capability, which may serve as a promising system for improving its applications in food and pharmaceutical products.

## 1. Introduction

Curcumin (CUR) is a hydrophobic polyphenol from the rhizome of the widely used turmeric spice (*Curcuma longa* L.) [1, 2]. It is a diarylheptanoid with two aromatic O‐methoxyphenolic groups connected by two *α*,*β*‐unsaturated carbonyl groups, chemically known as 1,7‐bis(4‐hydroxy‐3‐methoxyphenyl)‐1,6‐heptadien‐3,5‐dione [3]. CUR has been widely used in food and pharmaceutical fields because of its diverse biological functions, including antioxidant [2], anti‐inflammatory [4], antimicrobial [5], anticancer [6], and cardioprotective activities as well as immune modulation [7] and potential for treating diabetes and obesity [8]. However, the water solubility of CUR is poor, and it is also very sensitive to various environmental conditions, such as light, heat, and oxygen, which limits its application in food and pharmaceutical fields [9].

Embedding hydrophobic compounds within amphiphilic molecules or complexes through noncovalent interactions is an effective way to improve their water solubility [10, 11]. Specifically, most macromolecules are composed of a variety of amphiphilic building blocks that can form highly ordered assemblies with other molecules through noncovalent interactions including electrovalent bonds, *π*–*π* stacking, hydrogen bonds, Van der Waals forces, and hydrophobic interactions [12]. The nature of amphiphilic macromolecules (especially proteins, polysaccharides, lipids, etc.) has been explored as effective self‐assembled nanocarrier delivery systems (including nanoparticles, nanoemulsions/microemulsions, liposomes, and nanogels) to improve the bioavailability and efficacy of CUR [13–17]. However, the carriers comprise a much larger proportion of their formulations in most macromolecule‐based delivery systems, thereby limiting their loading capacity [18]. Therefore, a delivery system with high loading capacity and water solubility is needed to further explore [19].

Recently, small molecule self‐assembled nanoparticle technology is regarded as an excellent candidate for use as a carrier in nanoscale delivery systems due to its numerous advantages, such as a loading capacity and a convenient preparation process, not requiring any carrier or excipient [20, 21]. Among them, with the advantages of being handy, excellent biocompatibility, and safety, natural product–based small molecule self‐assembly has attracted much more attention [22]. Numerous studies have shown that CUR can self‐assemble with some natural products to form nanoparticles, improving water solubility and bioavailability [23, 24]. A combination of CUR and berberine (a quaternary alkaloid derived from doesoquinolines) demonstrated synergistic antimicrobial actions [25]. Zhang et al. also reported that a rubusoside‐solubilized CUR nanoparticle could increase the water solubility of CUR up to 2.318 mg/mL [20]. Interestingly, Wong et al. found that monosaccharide fructose could guide the self‐assembly of CUR to form 100–150 nm nanoparticles [21]. The self‐assembly of small molecules with CUR to obtain nanoparticle delivery systems will provide new avenues for their application in the food industry [26]. However, most small molecules used for CUR self‐assembly cannot be directly added to food. Therefore, finding food additives that can self‐assemble with CUR is of great significance for the application of CUR.

Mogrosides (MOGs) are cucurbitane‐type triterpene glycosides produced by monk fruit (*Siraitia grosvenorii*), composed of MOGs I–V, which have been granted GRAS (generally recognized as safe) nonnutritive sweetness by the US Food and Drug Administration [27]. MOGs have a hydrophobic side composed of coplanar aromatic rings and a hydrophilic side composed of glycoside bonds. The unique amphiphilic molecular structure makes it a candidate molecule for self‐assembly. Recently, it was reported that MOG V could assemble with CUR by using the solvent evaporation method, which increased the water solubility of CUR almost 6000 times [28]. However, the solvent evaporation method requires a large amount of organic solvents, which have potential toxicity and can cause environmental pollution.

Here, curcumin–mogroside nanoparticles (CUR–MOG NPs) were prepared by using a dialysis method. The structural properties of assembled CUR–MOG NPs were examined by using dynamic light scattering (DLS), transmission electron microscopy (TEM), x‐ray diffraction (XRD), UV‐visible spectrophotometry, Fourier transform infrared (FTIR) spectroscopy, and thermogravimetric analysis (TGA). Furthermore, the antioxidant and HepG2 cell cytotoxicity activities of CUR–MOG NPs were also evaluated.

## 2. Materials and Methods

### 2.1. Materials

The MOGs were purchased from SXBC Kangze Biotech Co. Ltd. (Xian, China), with the MOG V and total MOG contents of 50.54% and 98.24%, respectively. CUR (98 wt% purity) was purchased from Macklin Inc. (Shanghai, China). 1,1‐Diphenyl‐2‐picryl‐hydrazyl (DPPH) was purchased from Shanghai Yuanye Bio‐Technology Co. Ltd. (Shanghai, China). The apoptosis and necrosis detection kit with YO‐PRO‐1 and propidium iodide (PI) fluorescent dyes, methylthiazolyldiphenyl‐tetrazolium bromide (MTT), and penicillin–streptomycin (P/S) solution was purchased from Beyotime Biotechnology (Shanghai, China). Dulbecco’s modified Eagle’s medium (DMEM) and fetal bovine serum (FBS) were purchased from Gibco (Grand Island, New York, United States). Ninety‐six–well microplates were purchased from Biosharp Biotechnology (Shanghai, China). The HepG2 human liver cancer cells were provided by the Cell Bank of the Chinese Academy of Sciences (Shanghai, China). All other reagents were purchased from Sinopharm Chemical Reagent Co. (Shanghai, China).

### 2.2. Self‐Assembly Formation

Self‐assembly CUR–MOG NPs were prepared by using dialysis methods as described previously by Tian et al. [25] with some modifications. Briefly, MOG (1000 mg) and CUR (9.0 mg) were dissolved in 17 mL of deionized water and 3 mL of ethanol, respectively. Then, CUR solution was added to MOG solution in a dropwise manner. The mixture was stirred for another 15 min at 250 rpm in the dark and then dialyzed against deionized water at 4°C for 12 h with a 2‐kDa molecular weight cut‐off. The unassembled CUR or other water‐insoluble components were removed by centrifugation (10,000 rpm, 20 min). The CUR–MOG NP solution was withdrawn, and an equal volume of ethanol was added. After filtration with a 0.45‐*μ*m polytetrafluoroethylene (PTFE) filter, the concentration of CUR in the CUR–MOG NPs was determined by using the HPLC method [25]. Essentia LC‐16 liquid chromatography system (Shimadzu Instrument Suzhou Co. Ltd., Suzhou, China) was employed to analyze CUR contents. Ten microliters of each sample was injected into a reversed‐phase C18 column (Welch Ultimate ODS‐3, 250 × 4.6 mm, 5 *μ*m, Welch Technology Shanghai Co. Ltd., Shanghai, China) and eluted with Solution A (0.5% acetic acid) and Solution B (acetonitrile) with a procedure: 0 min 56% Solution A and 44% Solution B, 9 min 48% Solution A and 52% Solution B, 15 min 38% Solution A and 62% Solution B, and 25 min 56% Solution A and 44% Solution B. The flow rate was 1 mL/min, and the detection wavelength of CUR was 425 nm. The CUR assembly rate (AR) was calculated according to the following equation:

AR=NtN0×100%,

where *N*_0_ represents the initial amount of CUR and *N*_*t*_ represents the amount of CUR retained in the dialysis bag.

The CUR–MOG NP solutions were lyophilized and stored at 4°C for future use.

### 2.3. Characterization of Self‐Assembly CUR–MOG NPs

#### 2.3.1. TEM Observation

The microstructural morphology of CUR–MOG NPs was assessed using TEM. Solutions of CUR–MOG NPs were appropriately diluted and negatively stained with phosphotungstic acid. The morphologies were analyzed with a HITACHI HT7800 transmission scanning electron microscope (HITACHI Instrument Co., Japan) operating at an accelerating voltage of 80 kV.

#### 2.3.2. Measurement of Particle Size Distribution and Surface Charge (DLS and Zeta)

The average diameter and polydispersity index (PDI) of the nanoparticle dispersions (100 *μ*g/mL) were measured using a Nano‐ZS 90 instrument (Malvern Panalytical, United Kingdom) as reported in previous studies [29].

#### 2.3.3. Thermal Analysis

CUR and MOG powers were mixed in a ratio of 0.9:100 (designated as CUR–MOG PMs), which was used as control. Thermal analysis was performed by TGA. The CUR, MOG, CUR–MOG PM, and CUR–MOG NP melting points were determined by analyzing endotherms obtained in HITACHI STA200 (HITACHI Instrument Co., Japan).

#### 2.3.4. XRD Assay

XRD analysis of all samples (CUR, MOG, CUR–MOG PMs, and CUR–MOG NPs) was performed at ambient temperature using the D8 x‐ray diffractometer system (Rigaku SmartLab 9 KW, Japan) and Cu K*α* radiation (generator setting: 40 kV and 40 mA). The data were collected at a 2°/min scanning speed, spanning a wide range of angles from 5° to 50° [30].

#### 2.3.5. FTIR Spectroscopy

The samples were ground into a fine powder and sifted through a 200‐mesh sieve. The resulting powders were then compressed into tablets. Attenuated total reflectance FTIR spectroscopic analysis was performed using a Thermo Scientific Nicolet iS20 spectrometer (Thermo Scientific, United States) covering a range from 4000 to 400/cm, with 32 scans taken [15].

#### 2.3.6. UV‐Visible Spectroscopy

A UV spectrophotometer (N4S, Shanghai Yidian Analytical Instruments & Analysis Co.) was used to measure the UV‐visible absorption spectra of CUR and CUR–MOG NPs dispersed in ethanol and MOG dispersed in water. UV‐visible absorption scanning was performed from 200 to 600 nm at a scanning speed of 0.1°/min. The absorbance range was set between 0.0 and 2.0 for each sample [31]. To make the data comparisons possible, all samples were maintained at the same concentration equivalents and instrument parameters.

#### 2.3.7. Solubility Measurement

The solubility of CUR and CUR–MOG NPs was determined following the method proposed by Zhang et al. [28]. Initially, CUR and CUR–MOG NPs were dissolved in deionized water in a flask, then mixed at 150 rpm at 37°C for 24 h, and subsequently centrifuged at 10,000 rpm at 4°C for 15 min. The supernatant was diluted with water to the desired concentration. The absorbance value at 425 nm was measured using a UV spectrophotometer (N4S, Shanghai Yidian Analytical Instruments & Analysis Co.), and the solubility was calculated based on the standard curve:

Y=0.19110.0059X−R2=0.998,

where *Y* represents the content of CUR and *X* represents the absorbance value of CUR.

#### 2.3.8. DPPH Scavenging Activity

Based on the described process, CUR–MOG NPs were assessed for their DPPH scavenging activity [32]. The CUR–MOG NPs were dissolved in deionized water and then further diluted to obtain a final concentration of CUR at 2.5, 5, 10, 15, 20, 25, 30, and 40 *μ*g/mL. Similarly, the concentration of MOG was adjusted to match that of MOG in the nanoparticles after dissolving it in deionized water. The DPPH scavenging activities were performed as follows: The sample solution was mixed with 1 mL of DPPH (0.1 mM) solution, vortexed thoroughly, and incubated for 30 min in the dark. The absorbance was measured at 517 nm against an ethanol blank. The control group consists of a sample solution of the same concentration and an equal volume of DPPH solution. The activity of CUR to scavenge the DPPH radical was calculated with the following equation:

DPPH scavenging activity%=A0−A2−A1A0×100%,

where *A*_0_ is the absorbance of the blank and *A*_2_ and *A*_1_ were the absorbance of the samples and samples’ blank, respectively. An analysis of the DPPH scavenging activity in relation to the CUR concentration was conducted over the entire range of test CUR concentrations. As a result of regression analysis, the CUR concentration that scavenges 50% of DPPH was defined as 50% inhibitory concentration (IC_50_).

### 2.4. Anticancer Activities

#### 2.4.1. Cell Culture

HepG2 liver carcinoma cells were cultured using DMEM supplemented with 10% FBS and 1% P/S and maintained in a humidified incubator at 37°C with 5% CO_2_.

#### 2.4.2. Cytotoxicity

The cytotoxicity of CUR, MOG, and CUR–MOG NPs against HepG2 cells was assessed using an MTT assay following the methodology outlined by Chen et al. [33]. HepG2 cells were cultured in an incubator with a 37°C and 5% CO_2_ atmosphere. The cells were seeded at a density of 8000 cells per well on a 96‐well microplate in growth medium. After a 24‐h incubation period, varying concentrations (2.5–40 *μ*g/mL) of MOG, CUR, and CUR–MOG NP samples were added and incubated for an additional 24 h. The culture medium was then substituted with serum‐free fresh medium supplemented with 0.5 mg/mL of MTT and incubated for 2 h at 37°C. Subsequently, 100 *μ*L of DMSO was introduced into each well to dissolve the MTT. The microplate was gently agitated to ensure complete dissolution of the blue crystals. Then, the absorbance at 570 nm was measured using a BioTek Synergy H1 microplate reader (BioTek, United States), and the cytotoxicity was calculated according to the following:

Cell viability=A2−A0A1−A0×100%,

where *A*_2_ is the absorbance of the wells containing CUR, MOG, and CUR–MOG NPs; *A*_1_ is the absorbance of the control wells; and *A*_0_ is the absorbance of the cells without treatment.

#### 2.4.3. Cell Apoptosis by Confocal Fluorescence Microscopy

HepG2 cells were seeded into confocal dishes at a density of 1 × 10^5^ cells/well and incubated for 24 h. The CUR, MOG, or CUR–MOG NPs were added to the wells to a final concentration of 40 *μ*g/mL. Following incubation at 37°C for 24 h, the samples were washed away using PBS three times. Then, the cells were mixed with 500‐*μ*L YPO‐1‐PI solution at 37°C for 20 min and observed using a confocal fluorescence microscope (Olympus, Japan).

### 2.5. Statistical Analysis

The experimental data were analyzed using SPSS 22.0 for variance (ANOVA). The results of the experiment were expressed as mean ± SD, and to analyze the significant differences between samples, Duncan’s test was used. All experiments were conducted in triplicates.

## 3. Results and Discussion

### 3.1. Self‐Assembly CUR–MOG NP Characterization

#### 3.1.1. Self‐Assembly Formation and Morphological Studies

The CUR–MOG NPs were prepared by using the dialysis method [25] with the final concentrations of MOG and CUR of 50 mg/mL and 450 *μ*g/mL, respectively. Due to its hydrophobicity, CUR was prone to form aggregates in water (shown in Figure 1a(1)), while CUR–MOG NPs showed an explicit and transparent solution (Figure 1a(2)), demonstrating a significant improvement in its water solubility. As an amphiphilic molecule, MOG may improve its water solubility by assembling with CUR [34]. The AR of CUR in CUR–MOG NPs was detected by HPLC, and it displays good embedding efficiency with a CUR AR of 86.35*%* ± 0.877*%*. As measured by DLS, CUR–MOG NPs had the mean particle size, PDI, and zeta potential of 59.96 ± 0.852 nm, 0.077 ± 0.013, and −15.27 ± 1.155 mV, respectively (Figure 1b). This indicates that the obtained nanoparticles had good stability [25]. The morphology of CUR–MOG NPs and free MOG was further observed by using TEM. CUR–MOG NPs showed as spherical nanoparticles (Figure 1c). Although MOG could also form particles, it had a smaller diameter than those of CUR–MOG NPs (Figure 1d). Furthermore, the diameter of the particles was smaller than those measured by DLS. It is possible that electron microscopy analyzes the air‐dried nanoparticles, whereas DLS assesses the wet (swollen) nanoparticles [35].

Figure 1(a) Photographs of the solutions of (1) CUR and (2) CUR–MOG NPs. (b) Particle size distribution of prepared CUR–MOG NPs measured by a laser particle size analyzer. TEM image of the (c) CUR–MOG NPs (scale bar: 500 nm) and (d) MOG (scale bar: 500 nm).

**Figure:**
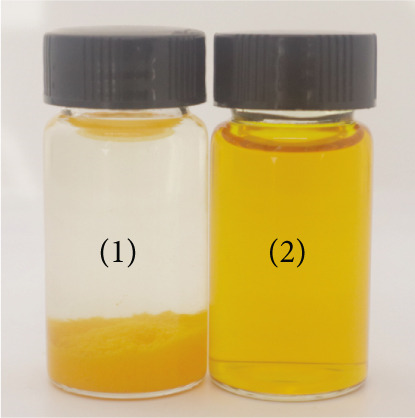
(a)

**Figure:**
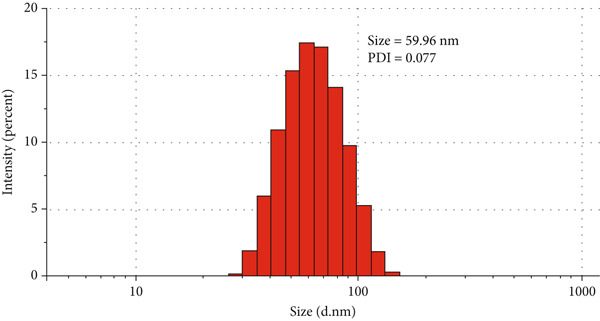
(b)

**Figure:**
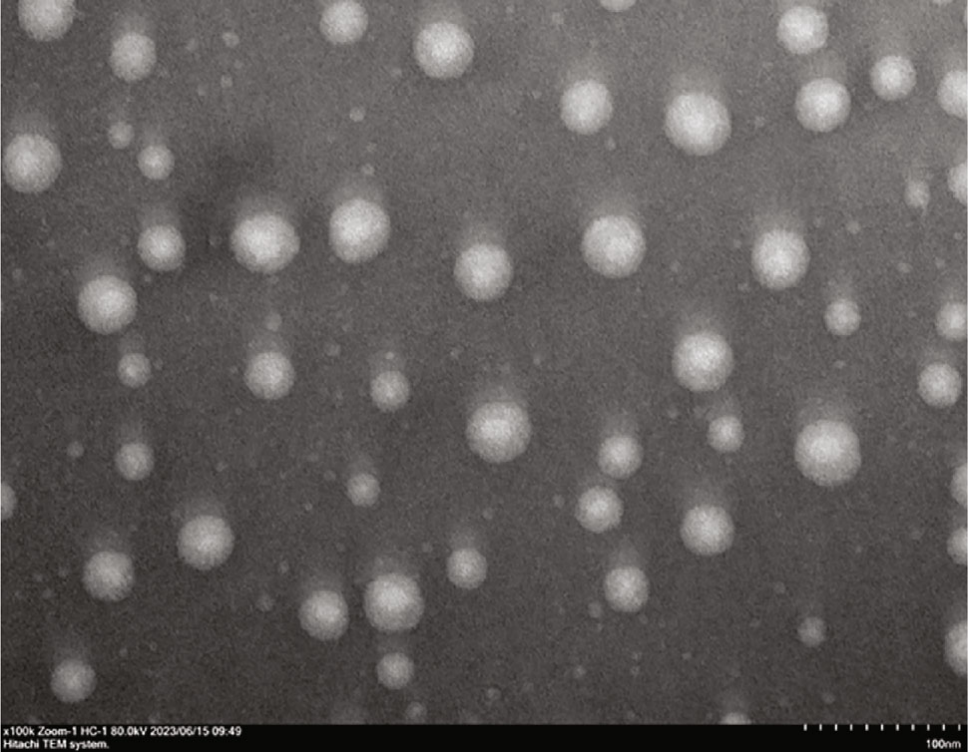
(c)

**Figure:**
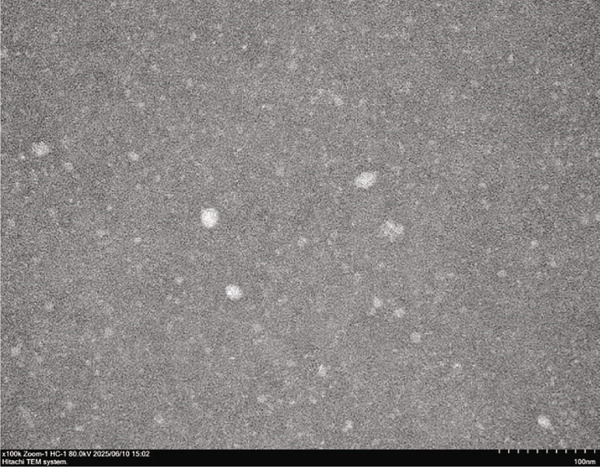
(d)

#### 3.1.2. Crystallinity of CUR, MOG, CUR–MOG PM, and CUR–MOG NPs

**Figure 2:**
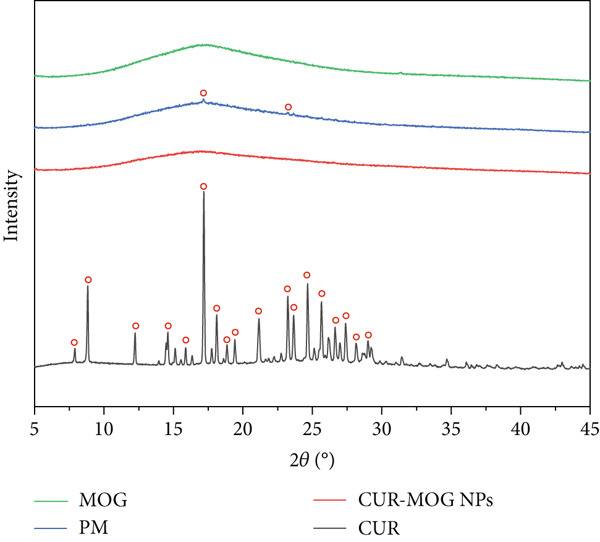
XRD patterns of CUR, CUR–MOG PM, MOG, and CUR–MOG NPs.

XRD was employed to validate the encapsulation of compound systems and crystalline changes of CUR [36]. As shown in Figure 2, CUR has characteristic absorption peaks at 8.798°, 17.172°, 18.109°, 12.214°, 14.552°, 23.267°, 24.269°, 25.680°, and 26.683°, which indicates that CUR is in a highly crystalline state as previously reported [37]. MOG shows broad amorphous bands. The characteristic absorption peaks of CUR appeared in CUR–MOG PM with a much weaker intensity, which may be due to the fact that the CUR to MOG rate was 0.9:100 in CUR–MOG PM. In contrast, the characteristic absorption peaks of CUR disappeared in CUR–MOG NPs (Figure 2). It was proposed that ingredients attached to each other in the complex may lead the stretching vibrations to disappear or not be apparent [38]. The XRD results suggested that CUR and MOG may form a new structure in CUR–MOG NPs. Moreover, although the CUR–MOG NPs showed a similar absorption peak profile to that of MOG, it had a weaker absorption intensity and a broader peak shape (Figure 2), which may be due to the interaction between CUR and MOG. CUR might be either trapped within the MOG in an amorphous form or in a solid‐state solubilized form [39]. The data definitively demonstrates that nanocomplexation with MOG caused CUR to undergo a transformation from a crystalline state to a microcrystalline or amorphous state.

#### 3.1.3. Spectral Characteristics

**Figure 3:**
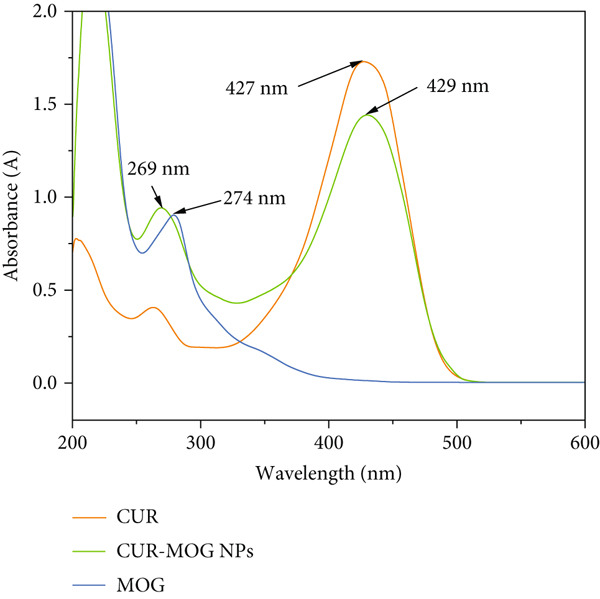
UV‐visible spectra of CUR, MOG, and CUR–MOG NPs.

UV‐visible spectroscopy was used to verify further insights into the interactions between CUR and MOG in CUR–MOG NPs by analysis of changes in the characteristic absorption peaks [31]. As shown in Figure 3, the maximum absorption (*λ*_max_) peak for MOG and CUR was 274 and 427 nm, respectively. The CUR–MOG NPs had similar *λ*_max_ absorption peaks to those of MOG and CUR but with a slight shift. The *λ*_max_ of MOG in CUR–MOG NPs showed a blue shift from 274 nm in free MOG to 269 nm, while the *λ*_max_ of CUR showed a slight red shift from 427 nm in free CUR to 429 nm. The noncovalent interaction, such as hydrogen bonds, *π*–*π* stacking, and hydrophobic interactions, can cause a red shift due to CUR assembly with other molecules [27, 32].

**Figure 4:**
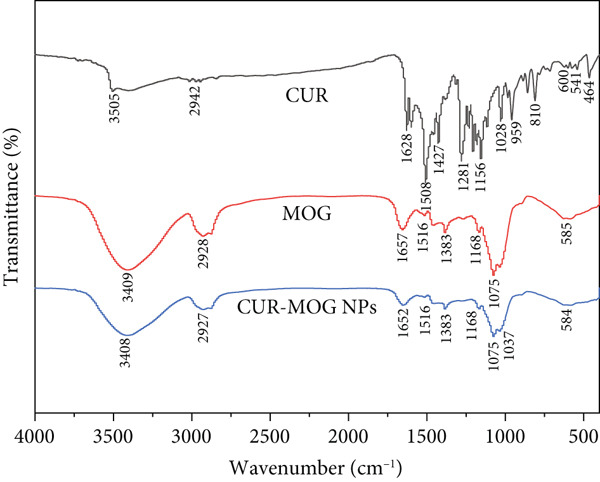
FTIR spectra of CUR, MOG, and CUR–MOG NPs.

FTIR spectroscopy was utilized to examine the specific interactions between CUR and MOG in the nanoparticles. The characteristic peaks of CUR were identified at 3505 cm^−1^ (phenolic O–H), 1628 cm^−1^ (C=O), 1508 cm^−1^ (C=C of benzene ring), 1281 cm^−1^ (aromatic hydrocarbon C–O), and 1156 and 1028 cm^−1^ for the stretching vibration of C–O–C [40], as illustrated in Figure 4. The prominent characteristic peaks of CUR diminish upon encapsulation in CUR–MOG NPs, particularly those associated with the benzene ring and aromatic hydrocarbon. This phenomenon can be attributed to the effective entrapment of the hydrophobic CUR within the interior of MOG through hydrogen bonding and hydrophobic interactions [35, 41]. Although CUR–MOG NPs and MOG had similar characteristic peaks, the intensity of CUR–MOG NP absorption peaks was much less than that of MOG. The characteristic peak of the carboxyl group (C=O) shifted from 1657 cm^−1^ in MOG to 1652 cm^−1^ CUR–MOG NPs, indicating more hydrogen bond perturbations among the materials [34].

#### 3.1.4. Thermal Analysis

The DSC method is employed to determine the melting and crystallization properties of CUR–MOG NPs [42]. The DSC curves of CUR, MOG, and CUR–MOG NPs are shown in Figure 5a. The DSC traces of CUR display a distinct endothermic peak at 183.3°C, signifying its *T*_m_ (melting temperature) [43]. This infers that CUR has a highly crystalline structure in its unconstrained state. The melting point for MOG was 73.7°C. However, the melting point of CUR–MOG NPs increased from 73.7°C to 74.3°C, which showed that the melting point is increased by the process of self‐assembly [44], indicating enhanced stability after self‐assembly with CUR.

Figure 5(a) DSC and (b) TG curves of CUR, MOG, and CUR–MOG NPs.

**Figure:**
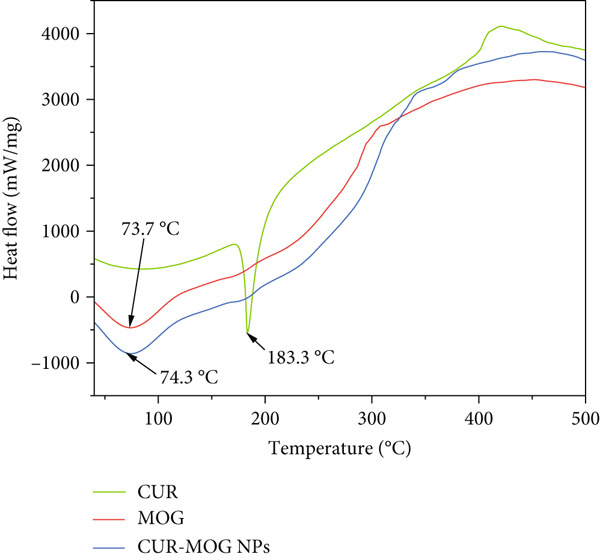
(a)

**Figure:**
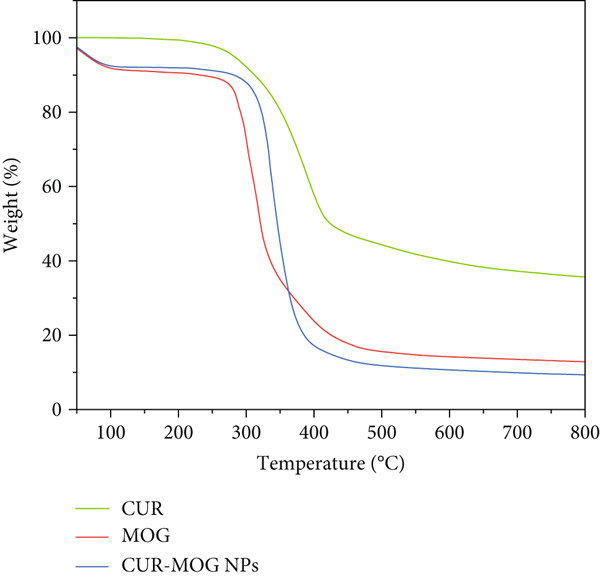
(b)

TG curves of CUR, MOG, and CUR–MOG NPs showed that CUR, MOG, and CUR–MOG NPs had 37%, 13%, and 9% residual matter at the end of thermal decomposition (Figure 5b), respectively, which was similar to the results of previous research studies [25, 45]. An explanation for this phenomenon might be the interaction between the two substances, causing amorphous carbon residues to be reduced during thermal decomposition [25].

### 3.2. The Water Solubility

The solubility of CUR and CUR–MOG NPs in water is presented in Figure 6. There was a noticeable difference in yellow color intensity between water solutions of CUR–MOG NPs and CUR (Figure 6a). The solubility of free CUR in water is 10.7 ng/mL (Figure 6b), which is similar to that previously reported by Kaminaga et al. [46]. In contrast, the water solubility of CUR–MOG NPs significantly increased up to 1.135 mg/mL (Figure 6b), making it approximately 10,606 times greater than that of free CUR. The increase in CUR water solubility was more pronounced compared to the method used by Zhang et al. [28]. It was also reported that rubusoside could lead to significantly increased CUR water solubility up to 2.318 mg/mL in the presence of 10% rubusoside [20]. The amphiphilic molecules self‐assemble with CUR through noncovalent interactions and can encapsulate it in the core of supermolecules, thereby improving the water solubility [25, 29]. Although the *π*–*π* stacking, hydrogen bonding, and hydrophobic interaction were proposed to contribute to CUR and MOG self‐assembly, further experiments are still needed to elucidate the mechanism in detail.

Figure 6The water solubility of CUR–MOG NPs. (a) Photographs of the solutions of (1) CUR and (2) CUR–MOG NPs. (b) Solubility of CUR and CUR–MOG NPs in water. Different letters (A, B) indicate significant differences among the different groups (*p* < 0.05).

**Figure:**
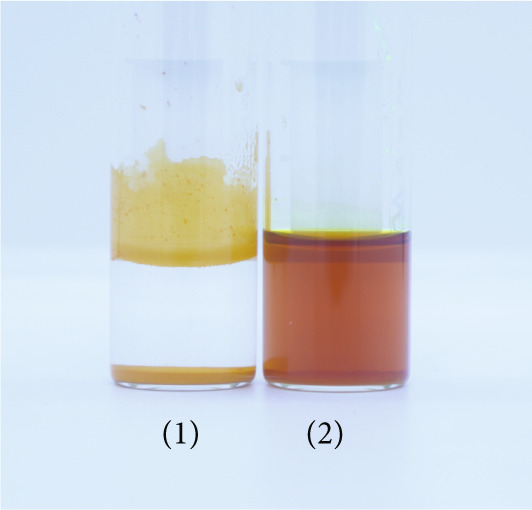
(a)

**Figure:**
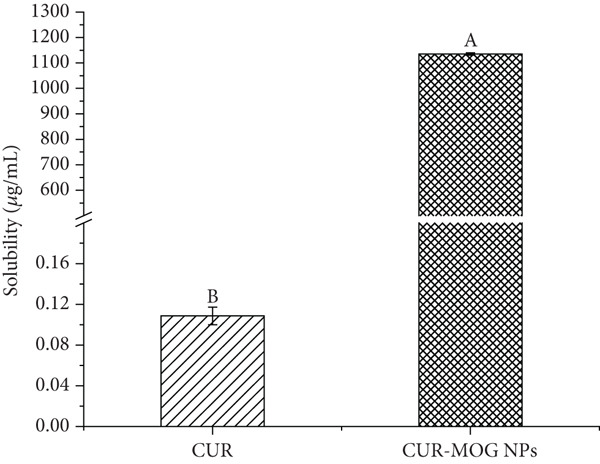
(b)

### 3.3. Antioxidant Capacity

The antioxidant activity of CUR, MOG, and CUR–MOG NPs was analyzed by evaluating the DPPH radical scavenging activity [47]. The DPPH radical scavenging activity of all samples was increased in a dose‐dependent manner (Figure 7). CUR and CUR–MOG NPs exhibited excellent antioxidant capacity (Figure 7a). The DPPH radical scavenging IC_50_ value of CUR and CUR–MOG NPs was 5.923 ± 1.130 and 5.576 ± 1.153 * μ*g/mL, respectively. In contrast, MOG showed the relatively low DPPH radical scavenging capacity (Figure 7b) with the IC_50_ value of 1.087 ± 0.681 mg/mL [48, 49]. The DPPH radical scavenging activity showed no differences between CUR and CUR–MOG NPs, indicating that the encapsulated CUR within MOG does not impact its antioxidant activity.

Figure 7DPPH radical scavenging activity of (a) CUR and CUR–MOG NPs and (b) MOG.

**Figure:**
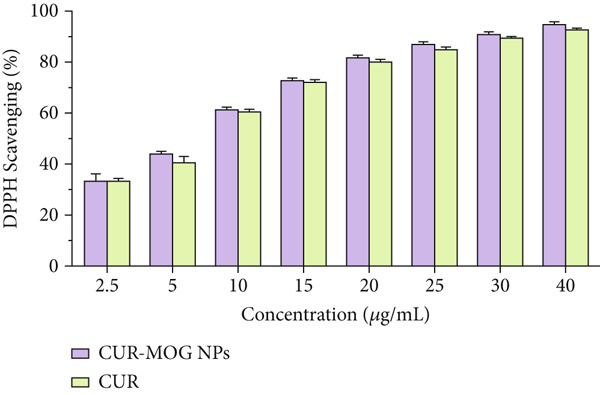
(a)

**Figure:**
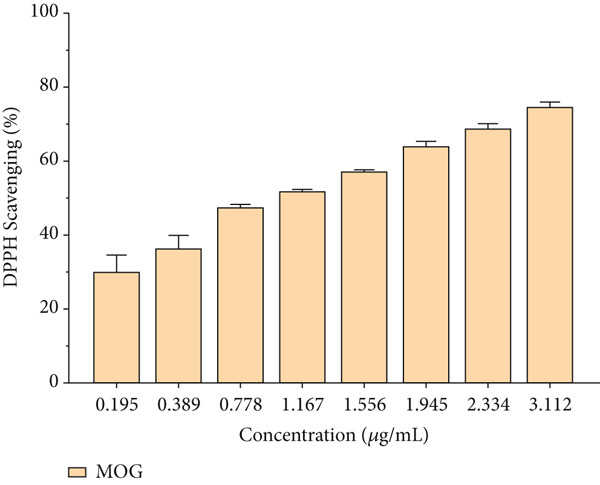
(b)

### 3.4. Cytotoxicity Analysis on HepG2 Cells

**Figure 8:**
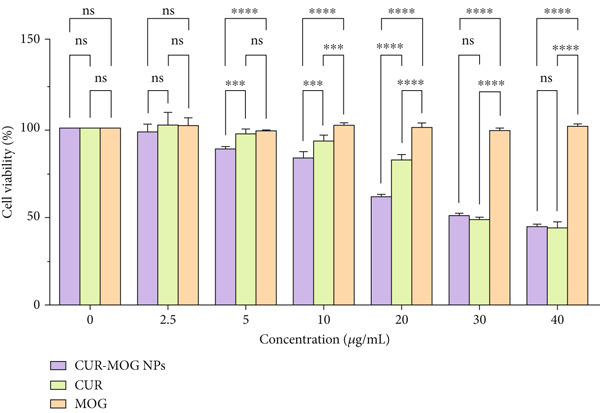
Effect of CUR, MOG, and CUR–MOG NPs on HepG2 cytotoxicity measured by MTT (3‐[4,5‐dimeylthiazol‐2‐yl]‐2,5‐diphenyltetrazolium bromide) after 24 h of incubation with various concentrations of test compound. Duncan’s test was used to analyze the significant differences among the different groups: ns, *p* > 0.05; ∗∗∗, *p* < 0.001; ∗∗∗∗, *p* < 0.0001.

CUR has been shown to reduce the viability of various types of cancer cells, such as those found in the stomach [50], colon [51], lung, liver [52], and breast [53]. To evaluate the influence of self‐assembly on its anticancer activity, the cytotoxicity of CUR, MOG, and CUR–MOG NPs to HepG2 liver carcinoma cells was examined. As shown in Figure 8, the cell viability (via MTT test) of HepG2 decreased along with the concentrations of CUR and CUR–MOG NPs increasing. The IC_50_ of CUR and CUR–MOG NPs was 33.91 ± 2.35 and 32.38 ± 1.255 * μ*g/mL, respectively. There was no statistically significant difference between the two groups (*p* < 0.05). Conversely, MOG did not have any inhibitory effect on HepG2 in the test concentration range. Although both CUR and CUR–MOG NPs demonstrated cytotoxicities toward HepG2, it can still be seen that at CUR concentrations < 20 * μ*g/mL, the cell viability of CUR–MOG NPs at any test concentration was significantly higher than that of CUR. In part, this may be due to the lipophilicity of CUR and its ability to pass through the cell membrane freely [6]. Therefore, encapsulated CUR may exhibit lower cytotoxicities due to its decreased mobility. It has been observed that CUR encapsulated in many types of polymeric particles or nanogels exhibits similar properties [54–56]. But when the CUR concentrations > 20 * μ*g/mL, the cell viability of CUR–MOG NPs was 51.733*%* ± 0.689*%* (at 30 *μ*g/mL) and 45.2059*%* ± 1.039*%* (at 40 *μ*g/mL), and that of CUR was 49.389*%* ± 1.504*%* (at 30 *μ*g/mL) and 44.709*%* ± 2.827*%* (at 40 *μ*g/mL); there was no statistically significant difference between the two groups (*p* < 0.05), indicating that both samples had equal inhibitory effects on HepG2 cells and the CUR anticancer activity does not diminish after the encapsulation of MOG.

**Figure 9:**
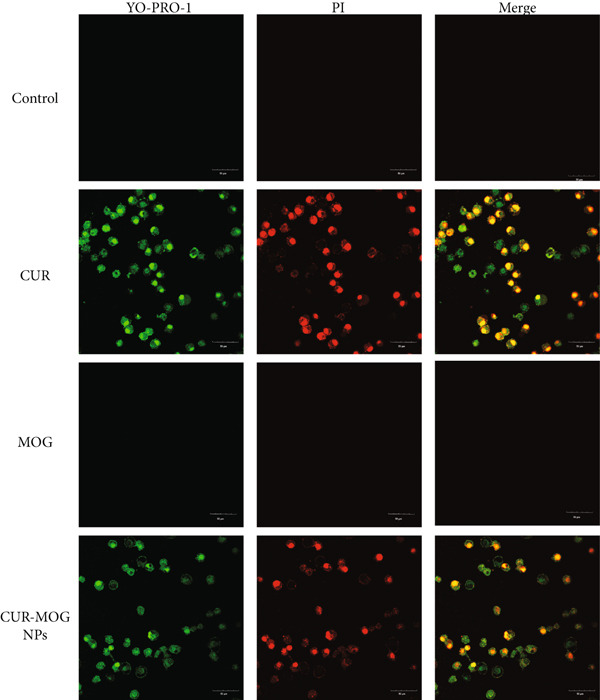
The effect of CUR, MOG, and CUR–MOG NPs on HepG2 cell apoptosis.

Many studies have proven that CUR induces cellular apoptosis to reduce viability [57, 58]. The effects of CUR–MOG NPs on the induction of HepG2 cell apoptosis were investigated by using the apoptosis and necrosis detection kit with YO‐PRO‐1 and PI. YO‐PRO‐1, also known as oxazole yellow, is a green fluorescent dye that can be used to detect apoptotic cells. It is nonpermeable to normal animal cell membranes but can penetrate the cell membranes of apoptotic cells. PI is a red fluorescent dye that binds to nucleic acids, emitting bright red fluorescence. It is used to stain only necrotic cells that have lost their cell membrane integrity. Untreated HepG2 cells were not stained by YO‐PRO‐1 as well as PI (Figure 9), but treatment with 40 *μ*g mL^−1^ of CUR or CUR–MOG NPs for 24 h could induce HepG2 cell apoptosis, and the nuclei of the apoptotic cells were stained with green fluorescence by YO‐PRO‐1. The necroptotic cell death was stained with both YO‐PRO‐1 and PI, with red and green fluorescence overlapping in an orange–yellow color. In contrast to CUR and CUR–MOG NPs, the MOG did not show any necroptotic cell death or apoptotic cell death signal. In brief, CUR–MOG NPs show a similar apoptosis induction activity to that of CUR, which was consistent with the results of MTT.

In conclusion, our study demonstrated that CUR–MOG NPs can effectively induce cell viability in HepG2 liver cancer cells in a concentration‐dependent manner. Moreover, the CUR–MOG NPs promoted the apoptosis of liver cancer cell lines.

## 4. Conclusion

The self‐assembly advantage lies in its predominantly water‐based composition with only a small component of organic solvent needed. Additionally, the assembly of the nanoparticle using this system can be performed without needing heat and in an open beaker. Here, the CUR–MOG NPs that were prepared displayed a particle size measuring 59.96 ± 0.852 nm, a low PDI of 0.077 ± 0.013, and a theta potential amounting to −15.27 ± 1.155 mV. SEM, DLS, XRD, FTIR, and TGA were used to determine successful self‐assembly of CUR–MOG NPs. The solubility of the CUR–MOG NPs was approximately 10,606 times greater than that of free CUR. The cell viability assays demonstrated the inhibitory effect of CUR–MOG NPs in liver cancer cell HepG2 lines. This study developed a new nanocarrier for insoluble polyphenols and expanded the nonnutritive sweetener applications as bioactive materials.

## Conflicts of Interest

The authors declare no conflicts of interest.

## Funding

No funding was received for this manuscript.

## Data Availability

The data that support the findings of this study are available from the corresponding author upon reasonable request.
